# RORA Regulates Autophagy in Hair Follicle Stem Cells by Upregulating the Expression Level of the *Sqstm1* Gene

**DOI:** 10.3390/biom15020299

**Published:** 2025-02-18

**Authors:** Xuefei Zhao, Yanchun Xu, Shuqi Li, Suying Bai, Wei Zhang, Yu Zhang

**Affiliations:** 1 College of Wildlife and Protected Area, Northeast Forestry University, Harbin 150040, China; zhaoxuefei@nefu.edu.cn (X.Z.);; 2 National Forestry and Grassland Administration Research Center of Engineering Technology for Wildlife Conservation and Utilization, Harbin 150040, China; 3 Detecting Center of Wildlife, State Forestry and Grassland Administration, Harbin 150040, China

**Keywords:** RORA, autophagy, hair follicle stem cells, hair follicle, *Sqstm1*

## Abstract

The hair coat is an adaptive evolutionary trait unique to mammals, aiding them in adapting to complex environmental challenges. Although some of the factors involved in regulating hair follicle development have been characterized, further in-depth research is still needed. Retinoic acid receptor-related orphan receptor alpha (RORA), as a member of the nuclear receptor family, is highly involved in the regulation of cellular states. Previous studies have shown that autophagy plays a significant role in hair follicle development. This study uses rat hair follicle stem cells (HFSCs) as a model to analyze the impact of RORA on the autophagy levels of HFSCs. Upon activation of RORA, autophagy indicators such as the LC3-II/LC3-I ratio and MDC staining significantly increased, suggesting an elevated level of autophagy in HFSCs. Following treatment with chloroquine, the LC3-II/LC3-I ratio, as well as the expression levels of BECN1 protein and SQSTM1 protein, were markedly elevated in the cells, indicating that the autophagic flux was unobstructed and ruling out the possibility that RORA activation impeded autophagy. Additionally, the level of the *Sqstm1* gene increased markedly after RORA activation promoted autophagy in the cells. We found that RORA regulates the transcription level of *Sqstm1* by binding to its promoter region. We believe that RORA activation significantly promotes the level of autophagy, particularly selective autophagy, in HFSCs, suggesting that RORA has the potential to become a new target for research on hair follicle development. This research provides a theoretical foundation for studies on hair follicle development and also offers new insights for the treatment of diseases such as alopecia.

## 1. Introduction

The hair coat is a unique keratinized skin appendage of mammals consisting of numerous independent hair follicle units. Based on morphological characteristics of hair follicles, they can be classified into primary and secondary hair follicles. The hair coat is a typical product of adaptive evolution, providing crucial functions such as insulation, sensation, and mechanical protection for animals to survive in harsh environmental conditions [1]. A typical hair follicle comprises microstructures such as the hair bulb, hair shaft, inner root sheath, arrector pili muscle, and sebaceous glands, with various cell types participating in the regulation of hair follicle development and homeostasis [2,3]. Hair follicles exhibit self-renewal properties, and a complete hair follicle cycle includes the anagen, catagen, and telogen phases [4,5]. An increasing number of studies are aiming to identify the driving factors of hair follicle renewal. In recent years, the role of autophagy in regulating hair follicle developmental status has gradually been unveiled. Studies have shown that both direct and indirect inhibition of the mTOR signaling pathway can induce autophagy to accelerate hair regeneration, while direct induction of autophagy through agonists can also promote hair growth. Furthermore, the level of autophagy during the anagen phase of the hair follicle cycle is higher than that in other phases, suggesting that autophagy promotes hair follicle development [6].

Autophagy is a physiological mechanism by which cells self-degrade and recycle their intracellular components. The classic autophagy model includes two crucial steps: the formation of autophagosomes that encapsulate the substrates to be degraded and the fusion of autophagosomes with lysosomes [7,8]. Autophagy can be classified based on its occurrence mode into macroautophagy, microautophagy, and chaperone-mediated autophagy; it can also be categorized based on the selectivity of degraded substrates into selective autophagy and non-selective autophagy [9,10,11]. Autophagy plays a dual role in the regulation of homeostasis: appropriate levels of autophagy help clear aged and useless intracellular substances, maintaining stability, whereas excessive autophagy may lead to over-clearance of cellular contents, thereby inhibiting cellular function [12,13,14,15]. Studies have shown that autophagy can affect the viability of hair follicle stem cells and promote hair regeneration by regulating glycolysis [16]. In addition, autophagy mediates cellular remodeling during the terminal differentiation of epidermal and skin keratinocytes [17]. Autophagy also plays a significant role in controlling stem cell activation induced by apoptosis and skin inflammation [18]. Evidence from organ cultures of human scalp hair follicles also indicates that autophagy is crucial in maintaining the regulatory processes of hair follicle development [19]. The impact of autophagy on hair follicle development also seems to exhibit duality, with studies showing both positive promotion of hair follicle development and negative effects on it [18]. This may be related to the specific developmental stage of the hair follicle and the particular cell types involved. Hair follicle stem cells are a type of adult stem cell. When hair follicles are in the catagen and telogen phases, hair follicle stem cells are mainly distributed in the bulge region where the arrector pili muscle intersects with the outer root sheath, and these stem cells are in a slow-cycling state. When hair follicles enter the anagen phase, hair follicle stem cells initiate proliferation and differentiation, migrating along the hair root sheath to complete the reconstruction of the hair follicle [20,21,22,23]. The cyclic activation of hair follicle stem cells is the driving force behind the cyclic reconstruction of hair follicles, and their characteristics make them the most important cell type in hair follicle development.

RORA is a ligand-dependent transcription factor that plays a crucial role in a series of physiological and pathological processes, including circadian rhythm regulation, metabolic regulation, inflammation, and immune system modulation [24,25,26,27]. When RORA was first discovered, its ligand was not clearly identified, and thus, it was classified as an orphan receptor. However, as related research has progressed, there is a perspective that RORA serves as the nuclear receptor for melatonin [28,29,30]. In recent years, there have been many differing views regarding the ligands of RORA. Some studies suggest that RORA may be a natural receptor for sterols and vitamin D derivatives [31]. RORA consists of a ligand-binding domain, a hinge region, and a DNA-binding domain. When activated by ligands, RORA can regulate the transcription levels of downstream target genes through monomeric or dimeric forms [32,33]. Fortunately, the discovery of synthetic agonists (such as SR1078) and inhibitors (such as SR3335) of RORA has greatly accelerated the progress in research on the functions and physiological effects of RORA. Studies have shown that RORA is highly correlated with conditions such as exercise, spatial cognition, and Parkinson’s disease [34,35,36]. Other studies have shown that RORA exhibits critical regulatory effects in cell apoptosis, epithelial–mesenchymal transition, and oxidative stress [37,38,39]. It is particularly noteworthy that RORA is considered to be highly involved in the regulation of circadian rhythms. Studies have shown that RORA affects circadian rhythms by regulating key circadian rhythm factors such as BMAL1 and CLOCK. Mice with RORA knockout exhibit significant disturbances in their metabolic circadian rhythms [40]. In cancer research, overexpression of the MYCN gene disrupts circadian rhythms and inhibits RORA expression. Restoring RORA activity or using RORA agonists can re-establish the cellular circadian clock [41]. Given the typical circadian rhythmicity of hair follicle development and the pivotal role of RORA in circadian regulation, studies have focused on the relationship between the two. In our preliminary research, we found that RORA may also be involved in regulating the autophagy levels of hair follicle stem cells. Therefore, this study uses rat hair follicle stem cells as a model to analyze the impact of RORA on their autophagy levels and attempts to characterize the underlying molecular regulatory mechanisms.

## 2. Materials and Methods

### 2.1. Cell and Drug Treatment

The cell model used in this study was primary hair follicle stem cells isolated from rat whiskers and long-term preserved in the laboratory. After 24 h of cell attachment and equilibration, the experimental group was treated with the RORA agonist SR1078 (MCE, Monmouth Junction, NJ, USA, HY-14422) at a final concentration of 10 μM, with a DMSO (1‰) control group set up. Chloroquine at 5 mM (MCE, HY-17589A) was used to block autophagy in cells to detect autophagic flux.

### 2.2. RNA Purification and Reverse Transcription

After treating the cells according to the experimental groups, the medium was removed, and the cells were washed three times with PBS buffer, with the supernatant removed as much as possible. RNA extraction from the cells was performed using the FastPure Cell/Tissue Total RNA Isolation Kit V2 (Vazyme, Nanjing, China, RC112-01). The Qubit 4 Fluorometer and Qubit™ RNA IQ Assay Kit (Invitrogen™, Carlsbad, CA, USA, Q33221) were used to assess RNA integrity. The PrimeScript™ RT reagent Kit with gDNA Eraser (Takara, Kyoto, Japan, RR047A) was used for reverse transcription to obtain cDNA. The steps and protocols for RNA extraction, quality assessment, and reverse transcription followed the instructions provided with the kits.

### 2.3. Quantitative Real-Time PCR (qRT-PCR)

The obtained cDNA samples were analyzed by qRT-PCR using the SsoAdvanced™ Universal SYBR^®^ Green (Bio-Rad, Hercules, CA, USA, 1725270) and the Bio-Rad CFX384 Real-Time PCR Detection System to detect the relative expression levels of the genes. The reaction system and program settings followed the instructions provided with the kit. Melting curve analysis was performed using the default program of the instrument. The Cyclophilin B (*Ppib*) gene was used as an internal reference gene for normalization of the results, and the 2^−ΔΔCt^ method was used for statistical analysis of gene differential expression levels. The primer sequences are provided in the Supplementary Materials.

### 2.4. Total Protein and Nuclear Protein Isolation

After drug treatment, the medium was removed from the cells, and 0.25% trypsin containing EDTA (Gibco, San Francisco, CA, USA, 25200056) preheated to 37 °C was added to digest the cells. Digestion was terminated when the cell connections became loose, and the cytoplasm contracted by adding a complete medium containing serum. The cell suspension was transferred to a centrifuge tube and centrifuged at 200 g for 5 min, with the supernatant removed. The cell precipitate was resuspended in PBS buffer and centrifuged again to thoroughly wash away any remaining liquid. RIPA lysis buffer containing 1 mM PMSF was added to the cell precipitate, and the mixture was pipetted and vortexed. After thorough mixing on a vortex mixer, the mixture was transferred to ice and left to stand for 10 min, with vortexing performed multiple times during this period to ensure complete cell lysis. After centrifugation again, the supernatant was collected and transferred to a new centrifuge tube as the total cellular protein. Nuclear protein extraction was performed using the NE-PER Nuclear and Cytoplasmic Extraction Reagents (Thermo Fisher, Waltham, MA, USA, 78833), with experimental steps following the instructions provided with the kit.

### 2.5. Western Blot

Protein samples were quantified using the BCA method, with the loading volumes adjusted to be consistent. Subsequently, 4× Protein SDS-PAGE Loading Buffer (Takara, Kyoto, Japan, 9173) was added in proportion. The samples were treated at 99 °C for 10 min to ensure complete denaturation of the protein samples. Electrophoresis was performed using a 4–20% gradient SDS-PAGE gel (Genscript, Piscataway, NJ, USA, M00655) to thoroughly separate the protein samples. After electrophoresis, the protein bands were transferred onto a PVDF membrane at 200 mA for 1 h. The PVDF membrane was then blocked with 5% BSA (in TBST) for 1 h to prevent non-specific binding. Following blocking, the membrane was incubated overnight at 4 °C with primary antibodies (Proteintech, Rosemont, IL, USA, 14600-1-AP, 11306-1-AP, 18420-1-AP, and 11607-1-AP. The dilution concentrations are 1:3000, 1:5000, 1:15,000, and 1:3000, respectively). The next day, after removing the primary antibodies, the PVDF membrane was thoroughly washed with TBST buffer three times for 10 min each. The membrane was then incubated with secondary antibodies (Proteintech SA00001-2. The dilution concentration is 1:5000.) for 1 h at room temperature. After another thorough washing with TBST buffer, the proteins were visualized using the ECL method (Meilunbio, Dalian, China, MA0186-1). Image acquisition was performed using the Bio-Rad ChemiDoc MP Imaging System, and grayscale analysis of the bands was conducted using ImageJ software v1.8.0.

### 2.6. Immunofluorescence

Cells were washed three times with PBS buffer for 3 min each. They were then fixed with 4% paraformaldehyde for 15 min. After removing the fixative, the cells were washed again three times with PBS buffer. The cells were permeabilized with 0.5% Triton X-100 for 20 min at room temperature, followed by three additional washes with PBS. The cells were then blocked with goat serum for 30 min at room temperature. Primary antibodies were added (SQSTM1 dilution is 1:750; BECN1 dilution is 1:200), and the cells were incubated overnight at 4 °C. The next day, after removing the primary antibodies, the cells were washed three times with PBS for 3 min each. Secondary antibodies were incubated for 1 h at room temperature, followed by washing with PBS buffer. The cells were mounted with an anti-fluorescence quenching mounting medium containing DAPI and observed under a fluorescence microscope for image acquisition.

### 2.7. MDC Detection

Cell autophagy levels were detected using the cell autophagy staining detection kit with MDC (monodansylcadaverine) (Beyotime, Shanghai, China, C3018S), following the instructions provided in the kit. Briefly, after removing the cell culture medium from different experimental groups, the cells were washed with PBS buffer. One milliliter of MDC staining solution was added, and the cells were incubated at 37 °C in the dark for 30 min. The MDC staining solution was removed, and the cells were washed three times with Assay Buffer. One milliliter of Assay Buffer was added, and the cells were observed under a fluorescence microscope.

### 2.8. Cleavage Under Targets and Release Using Nuclease

The Cleavage Under Targets and Release Using Nuclease (CUT&RUN) technique was employed to enrich downstream target gene fragments bound by RORA. The experiment was conducted using the CUT&RUN Assay Kit (Cell Signaling Technology, Danvers, MA, USA, #86652) and DNA Purification Kit (Cell Signaling Technology, #14209), with an IgG control group set up. The steps followed the instructions provided in the kits. The enriched DNA samples from the RORA target gene binding regions were then subjected to droplet digital PCR for detection of the *Sqstm1* promoter region.

### 2.9. Droplet Digital PCR

After appropriate dilution, samples were subjected to ddPCR using EvaGreen Digital PCR Supermix (Bio-Rad, CA, USA, #1864034). The reaction system and protocol were as specified in the kit’s instructions. A brief outline of the experimental procedure is as follows: the prepared PCR reaction mixture (20 μL) was transferred to a droplet generation card, and droplet generation oil (70 μL; Bio-Rad, #1864005) was added to the card. Water-in-oil droplets were generated using a droplet generator (Bio-Rad, #1864002) and transferred to a 96-well plate for PCR amplification. After the PCR reaction, droplets were read using a droplet reader (Bio-Rad, #1864003), and the experimental results were analyzed using Bio-Rad QuantaSoft™ Analysis Pro (QuantaSoft AP) softwareversion 1.4. Samples with more than 10,000 valid droplets analyzed were considered qualified for analysis.

### 2.10. Super-Shift Electrophoretic Mobility Shift Assay

Firstly, the motif structure of RORA-binding target genes was obtained from the JASPAR database, and a common specific sequence within these motifs was identified. This sequence was considered as the conserved region recognized and bound by the DNA-binding domain of the RORA protein. Probes targeting this region were designed and subjected to appropriate chemical modifications. Both wild-type and mutant probes were labeled with biotin, while cold probes (identical in sequence to the wild-type probes) were unlabeled. Electrophoretic Mobility Shift Assay (EMSA) analysis was conducted using the LightShift Chemiluminescent EMSA Kit (Thermo Fisher, 20148). A 6.5% native polyacrylamide gel was used for the separation of complexes, and a 0.45 μm pore size nylon membrane was used for blot transfer. The bands were visualized using the ECL method, and images were captured using the Bio-Rad ChemiDoc MP Imaging System. Steps followed the instructions provided in the kit.

### 2.11. Statistics

Each experiment included at least three biological replicates, and Student’s t-tests were used to assess statistical significance. All statistical analyses were performed using GraphPad Prism 9.5.1 software, and the results were expressed as mean ± standard deviation. *p* < 0.05 was considered to indicate a statistically significant result.

## 3. Result

### 3.1. SR1078-Induced RORA Activation Upregulates Autophagy Levels in HFSCs

**Figure 1:**
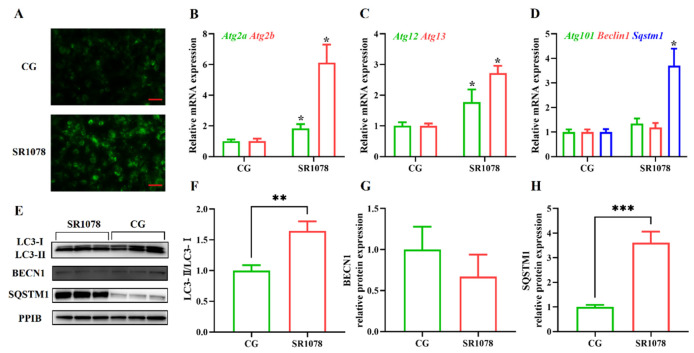
The impact of RORA activation on autophagy markers in HFSCs. * *p* < 0.05, ** 0.001 < *p* < 0.01, *** 0.0001 < *p* < 0.001. (**A**) Detection of cellular autophagy levels between the control group (CG) and the experimental group (treated with SR1078) using MDC staining. Scale bar, 25 μm. (**B**) Relative mRNA expression levels of *Atg2a* and *Atg2b* genes. (**C**) Relative mRNA expression levels of *Atg12* and *Atg13* genes. (**D**) Relative mRNA expression levels of *Atg101*, *Beclin 1*, and *Sqstm1* genes. (**E**) The WB detection of autophagy marker protein level changes between the control group and the experimental group (treated with SR1078) (n = 3). (**F**) Comparison of LC3-II/LC3-I levels between the control group (CG) and the experimental group (treated with SR1078). (**G**) Comparison of BECN1 levels between the control group (CG) and the experimental group (treated with SR1078). (**H**) Comparison of SQSTM1 levels between the control group (CG) and the experimental group (treated with SR1078). Original images can be found in Figure S1.

To analyze the impact of RORA on autophagy levels in HFSCs, we first treated the cells with SR1078, an agonist of RORA, and then detected changes in cellular autophagy levels using MDC staining. Experimental results showed that after activating RORA with SR1078, the autophagy levels of HFSCs significantly increased (Figure 1A), suggesting a positive correlation between RORA activation and cellular autophagy levels. Furthermore, we used qPCR to detect changes in mRNA levels of genes involved in the autophagy process. The results revealed that after activating RORA with SR1078, the transcription levels of *Atg2a* and *Atg2b* genes were upregulated 1.84-fold and 6.12-fold (Figure 1B), respectively, compared to the control group, while the transcription levels of *Atg12* and *Atg13* genes increased by 1.77-fold and 2.72-fold (Figure 1C), respectively. Although the transcription levels of *Beclin 1* and *Atg101* genes increased, the differences were not statistically significant (Figure 1D). Interestingly, the transcription level of the *Sqstm1* gene was also upregulated, approximately 3.7-fold compared to the control group (Figure 1D).

**Figure 2:**
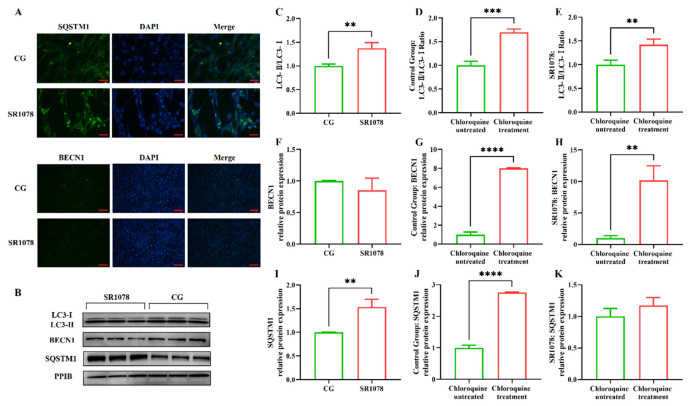
Evaluation of the impact of RORA activation on autophagy flux in HFSCs. ** 0.001 < *p* < 0.01, *** 0.0001 < *p* < 0.001, **** *p* < 0.0001. (**A**) The IF detection results of SQSTM1 (Scale bar, 25 μm) and BECN1 (Scale bar, 50 μm) between the control group (CG) and the experimental group (treated with SR1078). (**B**) The WB detection results of the differences in autophagy indicator proteins between the control group and the experimental group (treated with SR1078) after treated with chloroquine. (**C**) Changes in the LC3-II/LC3-I ratio in the experimental group (treated with SR1078) and control group after chloroquine treatment. (**D**) Changes in the LC3-II/LC3-I ratio before and after chloroquine treatment in the control group. (**E**) Changes in the LC3-II/LC3-I ratio before and after chloroquine treatment in the experimental group (treated with SR1078). (**F**) Changes in the BECN1 protein expression in the experimental group (treated with SR1078) and control group after chloroquine treatment. (**G**) Changes in the BECN1 protein expression before and after chloroquine treatment in the control group. (**H**) Changes in the BECN1 protein expression before and after chloroquine treatment in the experimental group (treated with SR1078). (**I**) Changes in the SQSTM1 protein expression in the experimental group (treated with SR1078) and control group after chloroquine treatment. (**J**) Changes in the SQSTM1 protein expression before and after chloroquine treatment in the control group. (**K**) Changes in the SQSTM1 protein expression before and after chloroquine treatment in the experimental group (treated with SR1078). Original images can be found in Figure S2.

After clarifying the regulatory role of RORA on the transcription levels of some autophagy-related genes in HFSCs, we further examined several important indicators of autophagy induction. Firstly, we analyzed LC3 protein levels using Western blotting (Figure 1E). The results showed that the ratio of LC3 II to LC3 I protein expression levels significantly increased after treating the cells with SR1078, with a 1.64-fold upregulation of the LC3 II/LC3 I ratio in HFSCs compared to the control group (Figure 1F). These results, along with MDC staining results, suggest that RORA activation induces an increase in autophagy levels in HFSCs. Subsequently, we performed WB to detect the expression levels of BECN1 and SQSTM1 proteins. The results showed that the changes in protein expression levels were consistent with the mRNA trends, with no significant difference in BECN1 protein expression before and after SR1078 treatment, but SQSTM1 protein expression levels increased by approximately 3.61-fold after SR1078 treatment (Figure 1G,H). Immunofluorescence results also indicated a significant increase in SQSTM1 protein levels after SR1078 treatment (Figure 2A).

The premise of using the LC3 II/LC3 I ratio to assess cellular autophagy levels is the unobstructed autophagic flux, especially when multiple test results consistently indicate a significant increase in the level of the *Sqstm1* gene, suggesting an impairment in autophagic flux. Therefore, we treated the cells with chloroquine to block the fusion of autophagosomes with lysosomes, allowing us to detect whether autophagic flux is normal. The results showed that the LC3 II/LC3 I ratio underwent significant changes before and after chloroquine treatment, with an increase observed in both the experimental and control groups, indicating that autophagic flux was unobstructed in both groups (Figure 2C–E). Compared to the control group, treatment with SR1078 had an impact on BECN1 protein levels, but the difference was not significant. However, after chloroquine treatment, BECN1 protein levels in both the experimental and control groups increased significantly compared to pre-treatment levels, suggesting that chloroquine-induced blockade of autophagic flux led to the accumulation of BECN1 protein. This indicates that autophagic flux was unobstructed before chloroquine treatment, while SR1078 had no significant effect on BECN1 protein levels. These findings are consistent with the LC3 II/LC3 I ratio results (Figure 2F–H). Additionally, SQSTM1 protein levels also changed before and after chloroquine treatment. Following autophagy blockade, SQSTM1 protein levels in the control group increased significantly, indicating that chloroquine treatment disrupted autophagic flux in the control group, leading to impaired SQSTM1 degradation and accumulation. In the experimental group treated with SR1078, SQSTM1 levels also increased significantly before and after chloroquine treatment, confirming that autophagic flux was not blocked by the experimental treatment (Figure 2I–K).

MDC staining results and changes in BECN1 and LC3 II/LC3 I ratios all demonstrate that SR1078 treatment leads to an increase in autophagy levels in HFSCs. However, this conclusion seems to contradict the changes in mRNA and protein levels of the *Sqstm1* gene. Generally, an increase in SQSTM1 protein levels is considered negatively correlated with autophagy levels. Therefore, we speculate that SR1078 treatment may have a specific impact on the transcription and expression of the *Sqstm1* gene.

### 3.2. RORA Binds to the Promoter Region of the Sqstm1 Gene

**Figure 3:**
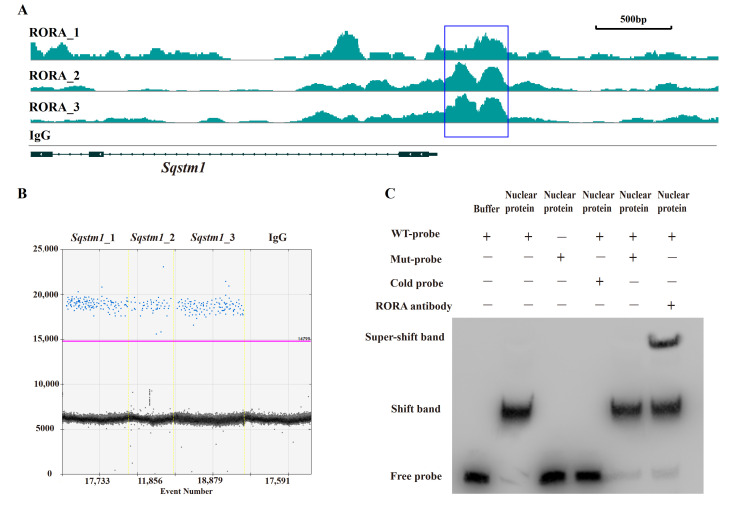
Verification of the interaction between RORA and the *Sqstm1* gene. (**A**) CUT&Tag enrichment signal in the promoter region of the *Sqstm1* gene [42]. (**B**) Detection results of ddPCR for fragments of the *Sqstm1* gene promoter region in CUT&RUN enrichment products. (**C**) The EMSA detection results for the interaction between RORA and *Sqstm1*. Original images can be found in Figure S3.

After elucidating the regulatory role of RORA on autophagy levels in HFSCs, we sought to analyze the underlying mechanisms, with particular attention to the abnormal changes in SQSTM1 protein levels. In our preliminary work using CUT&Tag, we analyzed potential downstream target genes of RORA and observed significant CUT&Tag positive signals in the promoter region of the *Sqstm1* gene. We obtained the known motif structure of potential RORA binding sites from the JASPAR database and identified a common sequence fragment, GGTCA. Notably, this common sequence was present in the CUT&Tag positive signal region within the *Sqstm1* gene promoter, located precisely at the center of the positive signal peak (Figure 3A). This suggests that RORA may bind to the *Sqstm1* gene promoter by recognizing and binding to this motif, thereby regulating the gene’s transcription level.

To further investigate, we enriched fragments bound to downstream target genes of RORA using RORA-specific antibodies and the CUT&RUN technique. To avoid false positives, we included a negative control using IgG antibodies. We designed specific primers targeting the promoter region of the *Sqstm1* gene around the GGTCA motif and detected the CUT&RUN enrichment products using ddPCR. The results showed positive signals in three parallel replicates but negative signals in the IgG negative control group (Figure 3B). This confirmed the binding of RORA to the promoter region of the *Sqstm1* gene, indicating that *Sqstm1* is likely a direct downstream target gene of RORA. Activated RORA directly participates in the regulation of *Sqstm1* gene transcription through this binding, which provides a plausible explanation for the seemingly contradictory phenomenon of significantly increased autophagy levels and SQSTM1 protein levels in HFSCs treated with SR1078 under conditions of unimpeded autophagy flux.

### 3.3. RORA Directly Binds to the Promoter Region of the Sqstm1 Gene

While the CUT&Tag and CUT&RUN techniques used in our previous and current studies demonstrate the binding of RORA protein to the *Sqstm1* gene promoter, they do not distinguish between direct and indirect binding. Therefore, we used EMSA to verify the direct binding of the RORA protein to the *Sqstm1* gene promoter. The experimental results indicate that in the negative control group, where biotin-labeled wild-type probes were present but no protein was added, only free probe bands were visible. However, when wild-type probes were incubated together with nuclear protein from HFSCs, a shift band was generated. When mutant probes were incubated together with nuclear protein, the shift band disappeared, and only free probe bands were present. In the competition experiment, we used excess unlabeled cold probes and biotin-labeled mutant probes to competitively inhibit the binding of wild-type probes to nuclear protein. The results indicated that excess mutant probes were unable to inhibit the formation of the shift band, whereas excess cold probes competitively inhibited the shift band. Additionally, when RORA protein-specific antibodies were added in addition to the wild-type probes during their incubation with nuclear protein, the formation of the super-shift band was observed (Figure 3C). These findings indicate that the RORA protein directly binds to the promoter region of the *Sqstm1* gene, recognizing specific motifs and directly regulating the transcription level of the *Sqstm1* gene.

## 4. Discussion

The periodic reconstruction of hair follicles in mammals is a typical phenomenon of organisms’ response to environmental stress [43]. Research on the developmental regulation of hair coat has been conducted for a long time, but progress has been slow, and we still know little about it. In particular, the inducing factors of hair follicle development or the transition between developmental stages deserve high attention. The factors regulating the periodic transition of HFSCs between activation and quiescence have been one of the research hotspots in this field in recent years. Autophagy, a conserved intrinsic homeostasis maintenance mechanism in organisms, is crucial in clearing aged and damaged proteins and organelles. Currently, the academic community is increasingly recognizing that autophagy may play a key regulatory role in hair follicle development. Numerous studies have analyzed the relationship between autophagy and hair follicle development, as well as the state of HFSCs [44,45,46]. Research shows that when photobiomodulation therapy is used to promote hair growth, enhanced autophagy levels during the anagen phase of hair follicles are one of the key factors promoting their regeneration [47]. Sun et al. found that induced autophagy promotes hair follicle development while blocking autophagy with 3-methyladenine leads to prolonged telogen and delayed anagen of hair follicles [16]. Meanwhile, exogenous activation of autophagy can initiate the anagen phase of mouse hair follicles and promote hair growth [6]. In addition, hair follicle development is regulated by multiple signaling pathways. Cai et al. found that the BMP and PTEN signaling pathways involved in hair follicle development regulate HFSC differentiation by modulating their autophagy levels [48]. Studies on the regulation of hair follicle development by Myristoleic acid (MA) have shown that MA promotes growth hormone signaling by activating the Wnt/β-catenin and ERK pathways in dermal papilla cells and regulating autophagy and cell cycle progression [49].

**Figure 4:**
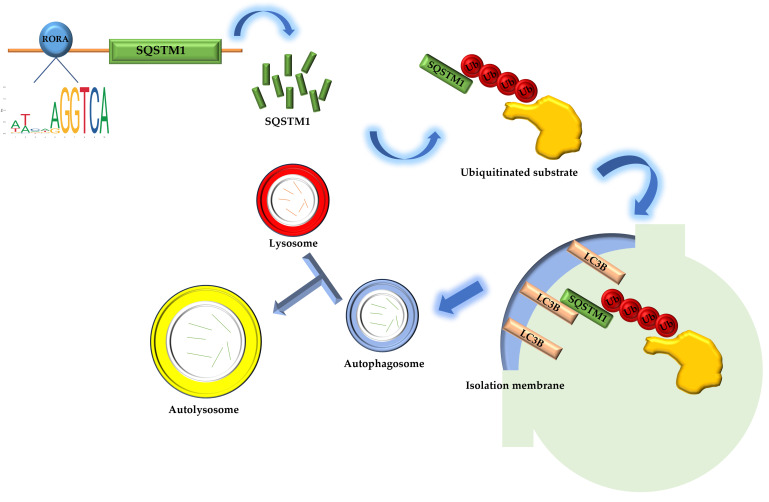
The schematic diagram of RORA involvement in the regulation of selective autophagy in hair follicle stem cells.

The *Sqstm1* gene plays a crucial role in various biological processes due to its complex domain structure. The SQSTM1 protein mediates phase separation through the polymerization of its N-terminal PB1 domain and the binding of its C-terminal UBA domain to ubiquitin [50]. In addition, the PB1 domain at the N-terminus of SQSTM1 may interact with Rpt1 and S5a/Rpn10 of the 26S proteasome, while the C-terminal UBA domain binds ubiquitinated proteins, facilitating the degradation of short-lived, misfolded, and damaged proteins through the ubiquitin–proteasome system. The SQSTM1 protein also undergoes continuous and rapid nucleocytoplasmic shuttling via its two nuclear localization signals (NLS1 and NLS2) and a nuclear export sequence (NES), enabling the transport of ubiquitinated cargo between the nucleus and cytoplasm [51,52]. In terms of oxidative stress regulation, the SQSTM1 protein modulates cellular oxidative stress by influencing the NRF2-KEAP1 signaling pathway [53,54,55,56]. Most importantly, the SQSTM1 protein is highly involved in selective autophagy [57]. The SQSTM1 protein binds to ubiquitin oligomeric chains of ubiquitinated substrates through its C-terminal UBA domain, while the PB1 domain assists in the packaging of ubiquitinated substrates through self-oligomerization. The LIR domain binds to the LC3B protein located on the isolation membrane, the precursor of autophagosomes, aiding the entry of ubiquitinated substrates into autophagosomes. Autophagosomes then fuse with lysosomes to form autolysosomes, ultimately completing the degradation of substrates [58]. Our research results indicate that activated RORA binds to the promoter region of the *Sqstm1* gene to regulate its transcription level. The upregulation of SQSTM1 protein expression suggests enhanced interactions between SQSTM1 adapter proteins, ubiquitinated substrates, and LC3B protein on the isolation membrane, thereby increasing the efficiency of ubiquitinated substrate entry into autophagosomes and ultimately upregulating the level of selective autophagy (Figure 4). The association between RORA and cellular autophagy has been demonstrated in related studies. During myocardial ischemia/reperfusion injury, cellular autophagy levels are significantly inhibited, and the absence of RORA significantly exacerbates this autophagy inhibition, suggesting that endogenous RORA may help restore autophagy function after myocardial ischemia/reperfusion injury [59]. Research in cancer also shows that RORA significantly promotes autophagy levels in cancer cells [60]. These findings corroborate the conclusion of our study that RORA promotes autophagy levels in HFSCs, but in previous studies focusing on the relationship between RORA and autophagy, the level of SQSTM1 protein was often considered to negatively correlate with the level of autophagy. However, our research has revealed that *Sqstm1* is a direct downstream target gene of RORA. Activation of RORA results in a significant upregulation of SQSTM1 levels in the hair follicle stem cells of rats. Meanwhile, changes in the LC3 II/LC3 I ratio and results from MDC staining all suggest an increase in autophagy levels. Therefore, we propose that RORA affects the autophagy level of HFSCs by upregulating selective autophagy.

RORA is recognized as a nuclear receptor for melatonin, which is an important molecule that transduces photoperiodic changes and has a well-established regulatory effect on the development of mammalian hair coats. This is also one of the important reasons why we focus on RORA [61,62,63,64,65,66]. Melatonin plays a crucial role in regulating biological rhythms as a bridge connecting photoperiodic signals and various physiological effects. The development of mammalian hair follicles exhibits obvious rhythmicity, further highlighting the importance of melatonin in hair follicle development. As the nuclear receptor for melatonin, RORA plays a vital role in mediating the physiological regulatory effects of melatonin. Studies have also shown that melatonin can regulate cellular autophagy levels through RORA [59,67,68]. Given the regulatory relationship between autophagy levels and the state of HFSCs and hair follicle development, we speculate that melatonin can regulate the autophagy levels of HFSCs through RORA, thereby affecting hair follicle development. In addition, as a transcription factor highly involved in the regulation of various cellular physiological states, RORA has the potential to become a new research target in studies related to hair follicle development. More research can be conducted on the regulatory effect of RORA on the autophagy levels of HFSCs, aiming to provide a theoretical basis for the molecular regulatory mechanisms of hair follicle development and offer new insights into research on the treatment of diseases such as alopecia.

## 5. Conclusions

In conclusion, this study found that activation of RORA upregulates the level of autophagy in rat hair follicle stem cells. Additionally, RORA can regulate the transcription level of the *Sqstm1* gene by directly binding to its promoter region, thereby suggesting an increase in selective autophagy levels. Furthermore, the elevation of selective autophagy induced by RORA may be the primary mechanism underlying its upregulation of autophagy in HFSCs.

## Data Availability

The original contributions presented in this study are included in the article/Supplementary Materials. Further inquiries can be directed to the corresponding author.

## Supplementary Materials

The following supporting information can be downloaded at https://www.mdpi.com/article/10.3390/biom15020299/s1. Figure S1: Original images of Figure 1E; Figure S2: Original images of Figure 2B; Figure S3: Original images of Figure 3C.

## References

[1] Du F., Li J., Zhang S., Zeng X., Nie J., Li Z. (2024). Oxidative stress in hair follicle development and hair growth: Signalling pathways, intervening mechanisms and potential of natural antioxidants. J. Cell. Mol. Med..

[2] Plowman J.E., Harland D.P., Plowman J.E., Harland D.P., DebChoudhury S. (2018). The Follicle Cycle in Brief. Hair Fibre: Proteins, Structure and Development.

[3] Schlake T. (2007). Determination of hair structure and shape. Semin. Cell Dev. Biol..

[4] Harland D.P., Plowman J.E., Harland D.P., DebChoudhury S. (2018). Environment of the Anagen Follicle. Hair Fibre: Proteins, Structure and Development.

[5] Lin X., Zhu L., He J. (2022). Morphogenesis, Growth Cycle and Molecular Regulation of Hair Follicles. Front. Cell Dev. Biol..

[6] Chai M., Jiang M., Vergnes L., Fu X., de Barros S.C., Doan N.B., Huang W., Chu J., Jiao J., Herschman H. (2019). Stimulation of Hair Growth by Small Molecules that Activate Autophagy. Cell Rep..

[7] DeMartino G.N. (2018). Introduction to the Thematic Minireview Series: Autophagy. J. Biol. Chem..

[8] Huang J., Brumell J.H. (2014). Bacteria-autophagy interplay: A battle for survival. Nat. Rev. Microbiol..

[9] Buzun K., Gornowicz A., Lesyk R., Bielawski K., Bielawska A. (2021). Autophagy Modulators in Cancer Therapy. Int. J. Mol. Sci..

[10] Ichimiya T., Yamakawa T., Hirano T., Yokoyama Y., Hayashi Y., Hirayama D., Wagatsuma K., Itoi T., Nakase H. (2020). Autophagy and Autophagy-Related Diseases: A Review. Int. J. Mol. Sci..

[11] Kang C., Elledge S.J. (2016). How autophagy both activates and inhibits cellular senescence. Autophagy.

[12] Ktistakis N.T., Tooze S.A. (2016). Digesting the Expanding Mechanisms of Autophagy. Trends Cell Biol..

[13] Li S., Wang L., Hu Y., Sheng R. (2015). Autophagy Regulators as Potential Cancer Therapeutic agents: A Review. Curr. Top. Med. Chem..

[14] Yim W.W.-Y., Mizushima N. (2020). Lysosome biology in autophagy. Cell Discov..

[15] Yun C.W., Jeon J., Go G., Lee J.H., Lee S.H. (2021). The Dual Role of Autophagy in Cancer Development and a Therapeutic Strategy for Cancer by Targeting Autophagy. Int. J. Mol. Sci..

[16] Sun P., Wang Z., Li S., Yin J., Gan Y., Liu S., Lin Z., Wang H., Fan Z., Qu Q. (2024). Autophagy induces hair follicle stem cell activation and hair follicle regeneration by regulating glycolysis. Cell Biosci..

[17] Eckhart L., Gruber F., Sukseree S. (2024). Autophagy—Mediated Cellular Remodeling during Terminal Differentiation of Keratinocytes in the Epidermis and Skin Appendages. Cells.

[18] Van Hove L., Toniolo A., Ghiasloo M., Lecomte K., Boone F., Ciers M., Raaijmakers K., Vandamme N., Roels J., Maschalidi S. (2023). Autophagy critically controls skin inflammation and apoptosis-induced stem cell activation. Autophagy.

[19] Parodi C., Hardman J.A., Allavena G., Marotta R., Catelani T., Bertolini M., Paus R., Grimaldi B. (2018). Autophagy is essential for maintaining the growth of a human (mini-)organ: Evidence from scalp hair follicle organ culture. PLoS Biol..

[20] Hoffman R.M. (2006). The pluripotency of hair follicle stem cells. Cell Cycle.

[21] Ji J., Ho B.S.-Y., Qian G., Xie X.-M., Bigliardi P.L., Bigliardi-Qi M. (2017). Aging in hair follicle stem cells and niche microenvironment. J. Dermatol..

[22] Joachimiak R., Bajek A., Drewa T. (2012). Hair follicle as a novel source of stem cells. Postep. Hig. I Med. Dosw..

[23] Ma D.R., Yang E.N., Lee S.T. (2004). A review: The location, molecular characterisation and multipotency of hair follicle epidermal stem cells. Ann. Acad. Med. Singap..

[24] Boukhtouche F., Doulazmi M., Frederic F., Dusart I., Brugg B., Mariani J. (2006). RORα, a pivotal nuclear receptor for Purkinje neuron survival and differentiation: From development to ageing. Cerebellum.

[25] Boukhtouche F., Mariani J., Tedgui A. (2004). The “CholesteROR” protective pathway in the vascular system. Arterioscler. Thromb. Vasc. Biol..

[26] Hu V.W. (2012). Is retinoic acid-related orphan receptor-alpha (RORA) a target for gene-environment interactions contributing to autism?. Neurotoxicology.

[27] Yemanyi F., Bora K., Blomfield A.K., Chen J. (2023). Retinoic Acid Receptor-Related Orphan Receptors (RORs) in Eye Development and Disease. Adv. Exp. Med. Biol..

[28] Dai J., Ram P.T., Yuan L., Spriggs L.L., Hill S.M. (2001). Transcriptional repression of RORalpha activity in human breast cancer cells by melatonin. Mol. Cell. Endocrinol..

[29] Lardone P.J., Guerrero J.M., Fernandez-Santos J.M., Rubio A., Martin-Lacave I., Carrillo-Vico A. (2011). Melatonin synthesized by T lymphocytes as a ligand of the retinoic acid-related orphan receptor. J. Pineal Res..

[30] Zhang Y., Zhao X., Li S., Xu Y., Bai S., Zhang W. (2024). Retinoic Acid-Related Orphan Receptor Alpha May Regulate the State of Hair Follicle Stem Cells by Upregulating the Expression of BNIP3. Animals.

[31] Slominski A.T., Kim T.-K., Takeda Y., Janjetovic Z., Brozyna A.A., Skobowiat C., Wang J., Postlethwaite A., Li W., Tuckey R.C. (2014). RORα and ROR γ are expressed in human skin and serve as receptors for endogenously produced noncalcemic 20-hydroxy- and 20,23-dihydroxyvitamin D. FASEB J..

[32] Dzhagalov I., Zhang N., He Y.-W. (2004). The Roles of Orphan Nuclear Receptors in the Development and Function of the Immune System. Cell. Mol. Immunol..

[33] Wada T., Kang H.S., Jetten A.M., Xie W. (2008). The emerging role of nuclear receptor RORα and its crosstalk with LXR in xeno- and endobiotic gene regulation. Exp. Biol. Med..

[34] Al-Zaid F.S., Hurley M.J., Dexter D.T., Gillies G.E. (2023). Neuroprotective role for RORA in Parkinson’s disease revealed by analysis of post-mortem brain and a dopaminergic cell line. NPJ Park. Dis..

[35] Caston J., Chianale C., Mariani J. (2004). Spatial memory of heterozygous staggerer (Rora+/Rora^sg^) versus normal (Rora^+^/Rora^+^) mice during aging. Behav. Genet..

[36] Caston J., Hilber P., Chianale C., Mariani J. (2003). Effect of training on motor abilities of heterozygous staggerer mutant (Rora^+^/Rora^sg^) mice during aging. Behav. Brain Res..

[37] Cai X., Zhang P., Wang S., Hong L., Yu S., Li B., Zeng H., Yang X., Shao L. (2020). lncRNA FGD5 antisense RNA 1 upregulates RORA to suppress hypoxic injury of human cardiomyocyte cells by inhibiting oxidative stress and apoptosis via miR-195. Mol. Med. Rep..

[38] Li D., Liu G., Wu Y. (2022). RORA alleviates LPS-induced apoptosis of renal epithelial cells by promoting PGC-1α transcription. Clin. Exp. Nephrol..

[39] Su J., Zhao X., Liu F., Xia H., Su B., Ling H., Zeng X., Su Q. (2017). Overexpression of RORa inhibits epithelial-mesenchymal transformation in human gastric cancer MGC803 cells. Chin. J. Clin. Exp. Pathol..

[40] Kojetin D.J., Burris T.P. (2014). REV-ERB and ROR nuclear receptors as drug targets. Nat. Rev. Drug Discov..

[41] Moreno-Smith M., Milazzo G., Tao L., Fekry B., Zhu B., Mohammad M.A., Di Giacomo S., Borkar R., Reddy K.R.K., Capasso M. (2021). Restoration of the molecular clock is tumor suppressive in neuroblastoma. Nat. Commun..

[42] Zhang Y., Zhao X., Li S., Xu Y., Bai S., Zhang W. (2025). Melatonin-Mediated Circadian Rhythm Signaling Exhibits Bidirectional Regulatory Effects on the State of Hair Follicle Stem Cells. Biomolecules.

[43] Mills K.K., Brandler O.V., Olson L.E. (2024). A review of molt in mammals, with an emphasis on marmots (Rodentia: Sciuridae: Marmota). J. Mammal..

[44] Du J., Liu W., Song Y., Zhang Y., Dong C., Xiong S., Huang Z., Wang T., Ding J., He Q. (2024). Activating autophagy promotes skin regeneration induced by mechanical stretch during tissue expansion. Burn. Trauma.

[45] Manzoor M., Chen D., Lin J., Wang Y., Xiang L., Qi J. (2025). Isoquercitrin promotes hair growth through induction of autophagy and angiogenesis by targeting AMPK and IGF-1R. Phytomedicine.

[46] Gund R., Christiano A.M. (2023). Impaired autophagy promotes hair loss in the C3H/HeJ mouse model of alopecia areata. Autophagy.

[47] Sun S.-Q., Shen J.-J., Wang Y.-F., Jiang Y.-T., Chen L.-F., Xin H., Wang J.-N., Shi X.-B., Zhu X.-Z., Sun Q. (2023). Red organic light-emitting diodes based photobiomodulation therapy enabling prominent hair growth. Nano Res..

[48] Cai B., Zheng Y., Yan J., Wang J., Liu X., Yin G. (2019). BMP2-mediated PTEN enhancement promotes differentiation of hair follicle stem cells by inducing autophagy. Exp. Cell Res..

[49] Choi Y.K., Kang J.-I., Hyun J.W., Koh Y.S., Kang J.-H., Hyun C.-G., Yoon K.-S., Lee K.S., Lee C.M., Kim T.Y. (2021). Myristoleic Acid Promotes Anagen Signaling by Autophagy through Activating Wnt/β-Catenin and ERK Pathways in Dermal Papilla Cells. Biomol. Ther..

[50] Sun D., Wu R., Zheng J., Li P., Yu L. (2018). Polyubiquitin chain-induced p62 phase separation drives autophagic cargo segregation. Cell Res..

[51] Pankiv S., Lamark T., Bruun J.-A., Overvatn A., Bjorkoy G., Johansen T. (2010). Nucleocytoplasmic Shuttling of p62/SQSTM1 and Its Role in Recruitment of Nuclear Polyubiquitinated Proteins to Promyelocytic Leukemia Bodies. J. Biol. Chem..

[52] Duran A., Amanchy R., Linares J.F., Joshi J., Abu-Baker S., Porollo A., Hansen M., Moscat J., Diaz-Meco M.T. (2011). p62 Is a Key Regulator of Nutrient Sensing in the mTORC1 Pathway. Mol. Cell.

[53] Ichimura Y., Waguri S., Sou Y.-s., Kageyama S., Hasegawa J., Ishimura R., Saito T., Yang Y., Kouno T., Fukutomi T. (2013). Phosphorylation of p62 Activates the Keap1-Nrf2 Pathway during Selective Autophagy. Mol. Cell.

[54] Liao W., Wang Z., Fu Z., Ma H., Jiang M., Xu A., Zhang W. (2019). p62/SQSTM1 protects against cisplatin-induced oxidative stress in kidneys by mediating the cross talk between autophagy and the Keap1-Nrf2 signalling pathway. Free. Radic. Res..

[55] Ho C.J., Gorski S.M. (2019). Molecular Mechanisms Underlying Autophagy-Mediated Treatment Resistance in Cancer. Cancers.

[56] de la Vega M.R., Chapman E., Zhang D.D. (2018). NRF2 and the Hallmarks of Cancer. Cancer Cell.

[57] Liu J., Kuang F., Kroemer G., Klionsky D.J., Kang R., Tang D. (2020). Autophagy-Dependent Ferroptosis: Machinery and Regulation. Cell Chem. Biol..

[58] Kraft L.J., Dowler J., Manral P., Kenworthy A.K. (2016). Size, organization, and dynamics of soluble SQSTM1 and LC3-SQSTM1 complexes in living cells. Autophagy.

[59] He B., Zhao Y., Xu L., Gao L., Su Y., Lin N., Pu J. (2016). The nuclear melatonin receptor ROR is a novel endogenous defender against myocardial ischemia/reperfusion injury. J. Pineal Res..

[60] Yan G., Lei H., He M., Gong R., Wang Y., He X., Li G., Pang P., Li X., Yu S. (2020). Melatonin triggers autophagic cell death by regulating RORC in Hodgkin lymphoma. Biomed. Pharmacother..

[61] Babadjouni A., Reddy M., Zhang R., Raffi J., Phong C., Mesinkovska N. (2023). Melatonin and the Human Hair Follicle. J. Drugs Dermatol..

[62] Fischer T.W. (2009). The influence of melatonin on hair physiology. Hautarzt.

[63] Fischer T.W., Slominski A., Tobin D.J., Paus R. (2008). Melatonin and the hair follicle. J. Pineal Res..

[64] Ibraheem M., Galbraith H., Scaife J., Ewen S. (1994). Growth of secondary hair follicles of the Cashmere goat in vitro and their response to prolactin and melatonin. J. Anat..

[65] Rong Y., Ma R., Zhang Y., Guo Z. (2024). Melatonin’s effect on hair follicles in a goat (*Capra hircus*) animal model. Front. Endocrinol..

[66] Yang C.H., Duan C.H., Wu Z.Y., Li Y., Luan Y.Y., Fu X.J., Zhang C.X., Zhang W. (2021). Effects of melatonin administration to cashmere goats on cashmere production and hair follicle characteristics in two consecutive cashmere growth cycles. Domest. Anim. Endocrinol..

[67] Chen Y., Zhang S.-P., Gong W.-W., Zheng Y.-Y., Shen J.-R., Liu X., Gu Y.-H., Shi J.-H., Meng G.-L. (2023). Novel Therapeutic Potential of Retinoid-Related Orphan Receptor α in Cardiovascular Diseases. Int. J. Mol. Sci..

[68] Zhao Y., Xu L., Ding S., Lin N., Ji Q., Gao L., Su Y., He B., Pu J. (2017). Novel protective role of the circadian nuclear receptor retinoic acid-related orphan receptor-α in diabetic cardiomyopathy. J. Pineal Res..

