# Supplementary figures and images for: RORA Regulates Autophagy in Hair Follicle Stem Cells by Upregulating the Expression Level of the Sqstm1 Gene
Source: Biomolecules. 2025 Feb 18;15(2):299. doi: 10.3390/biom15020299 (PMC11853448; doi:10.3390/biom15020299)

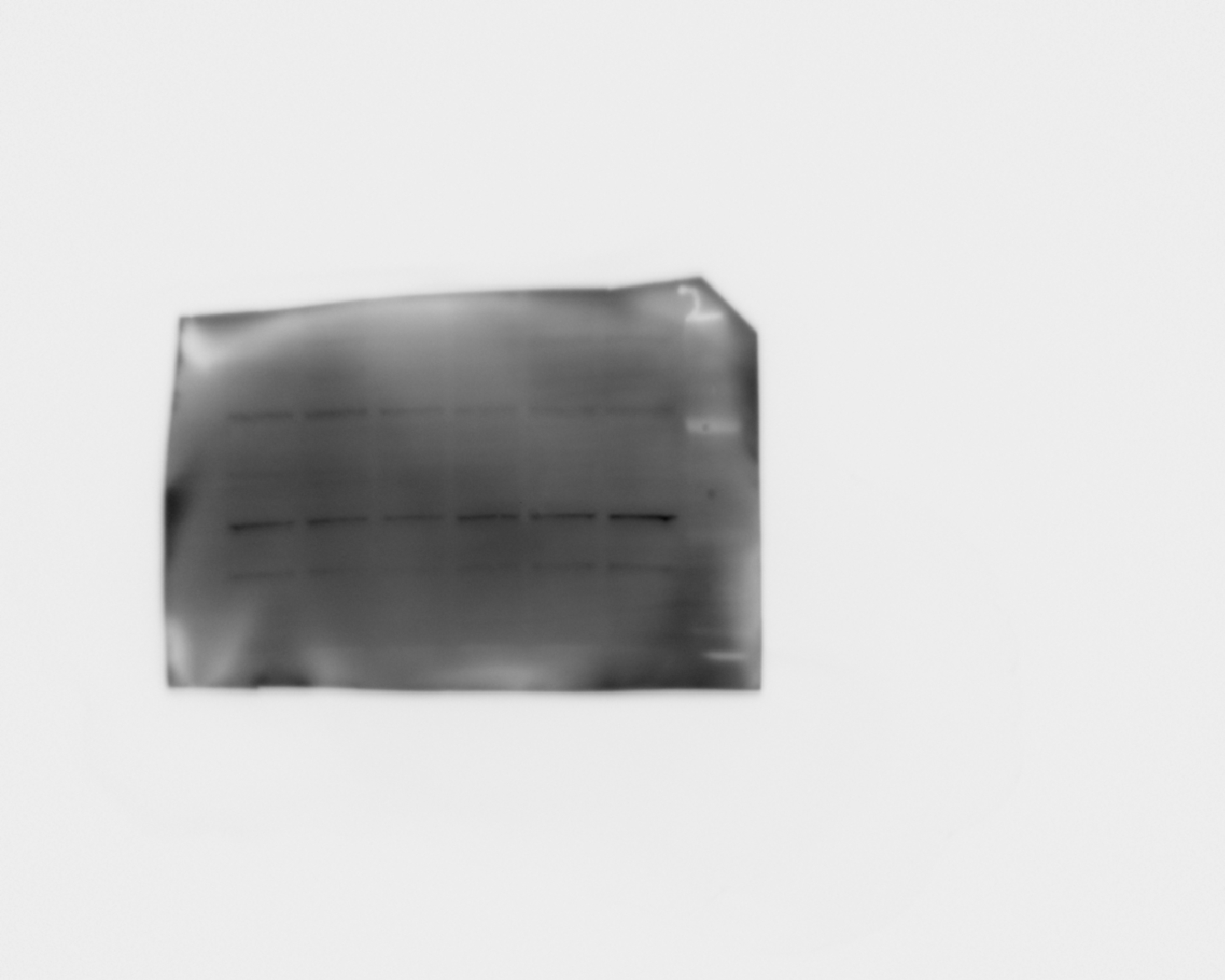

Supplement: Supplementary file 1 [file biomolecules-15-00299-s001.zip › File S2 Uncropped Images/Figure S1-BECN1.tif]

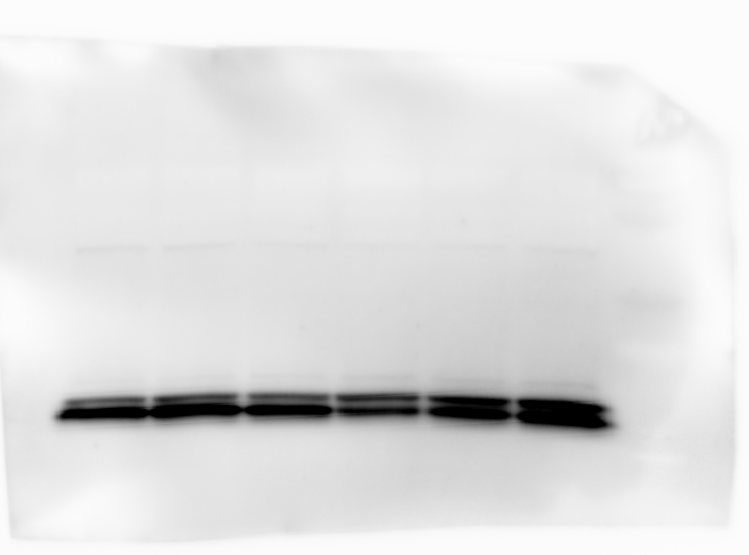

Supplement: Supplementary file 1 [file biomolecules-15-00299-s001.zip › File S2 Uncropped Images/Figure S1-LC3.tif]

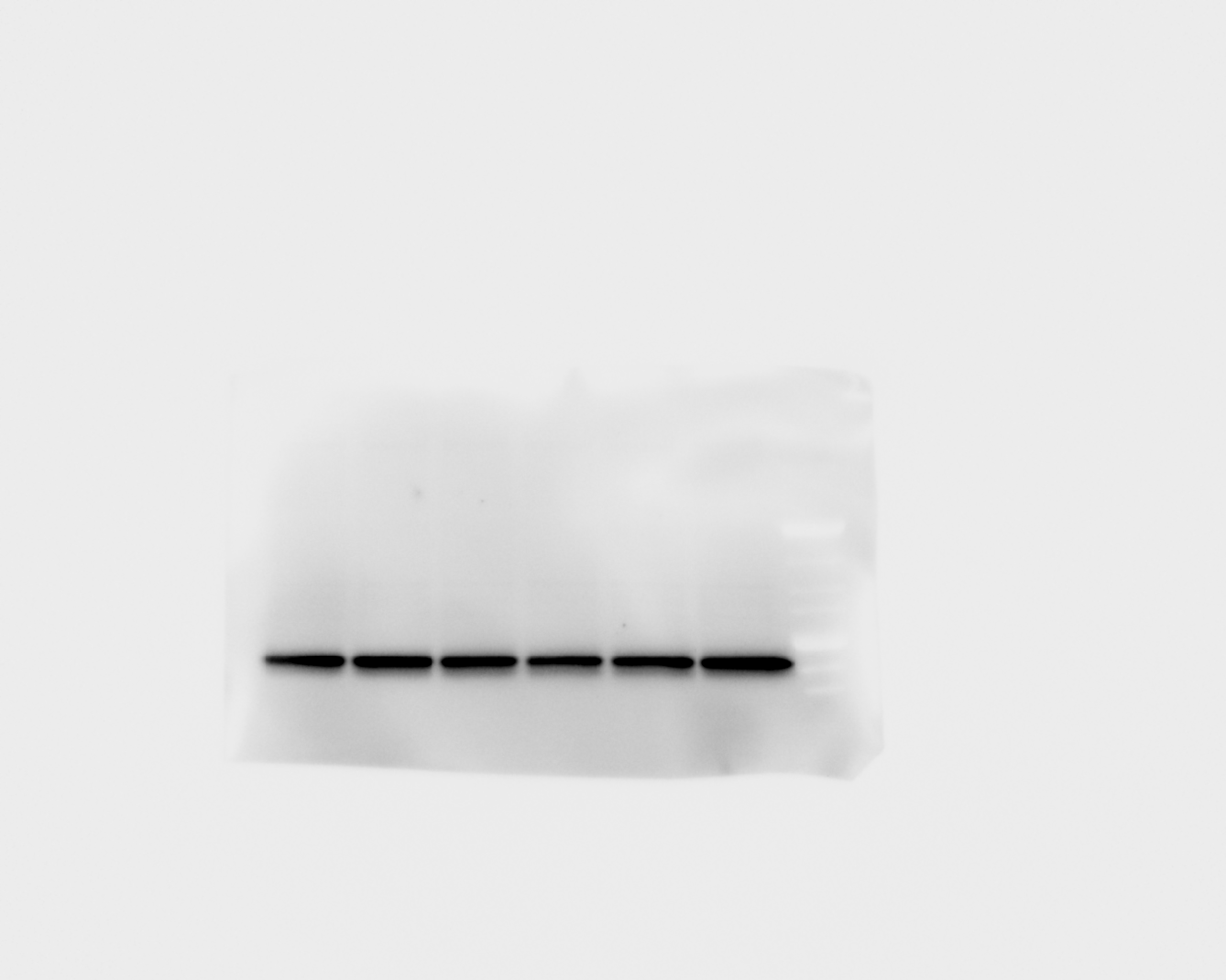

Supplement: Supplementary file 1 [file biomolecules-15-00299-s001.zip › File S2 Uncropped Images/Figure S1-PPIB.tif]

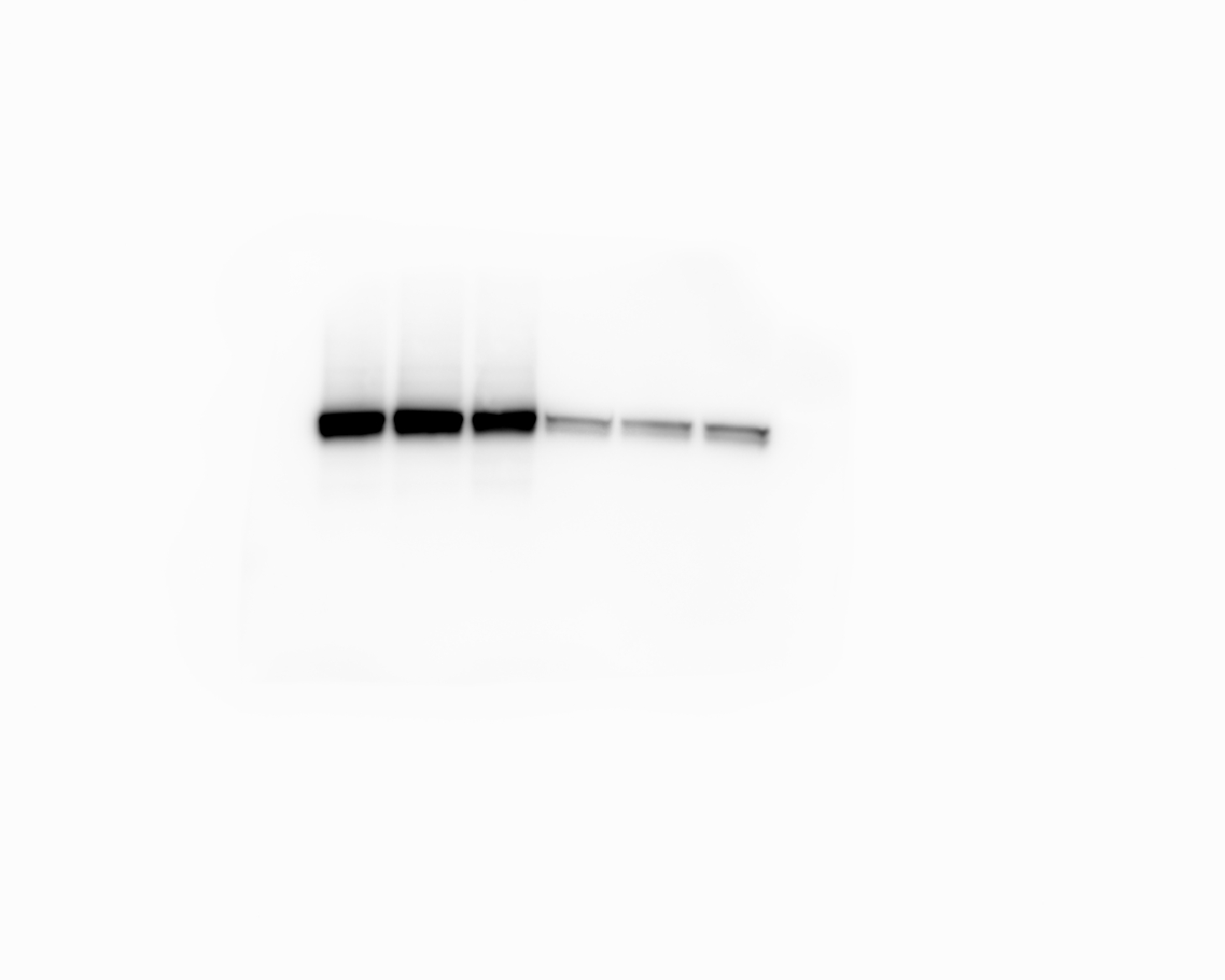

Supplement: Supplementary file 1 [file biomolecules-15-00299-s001.zip › File S2 Uncropped Images/Figure S1-SQSTM1.tif]

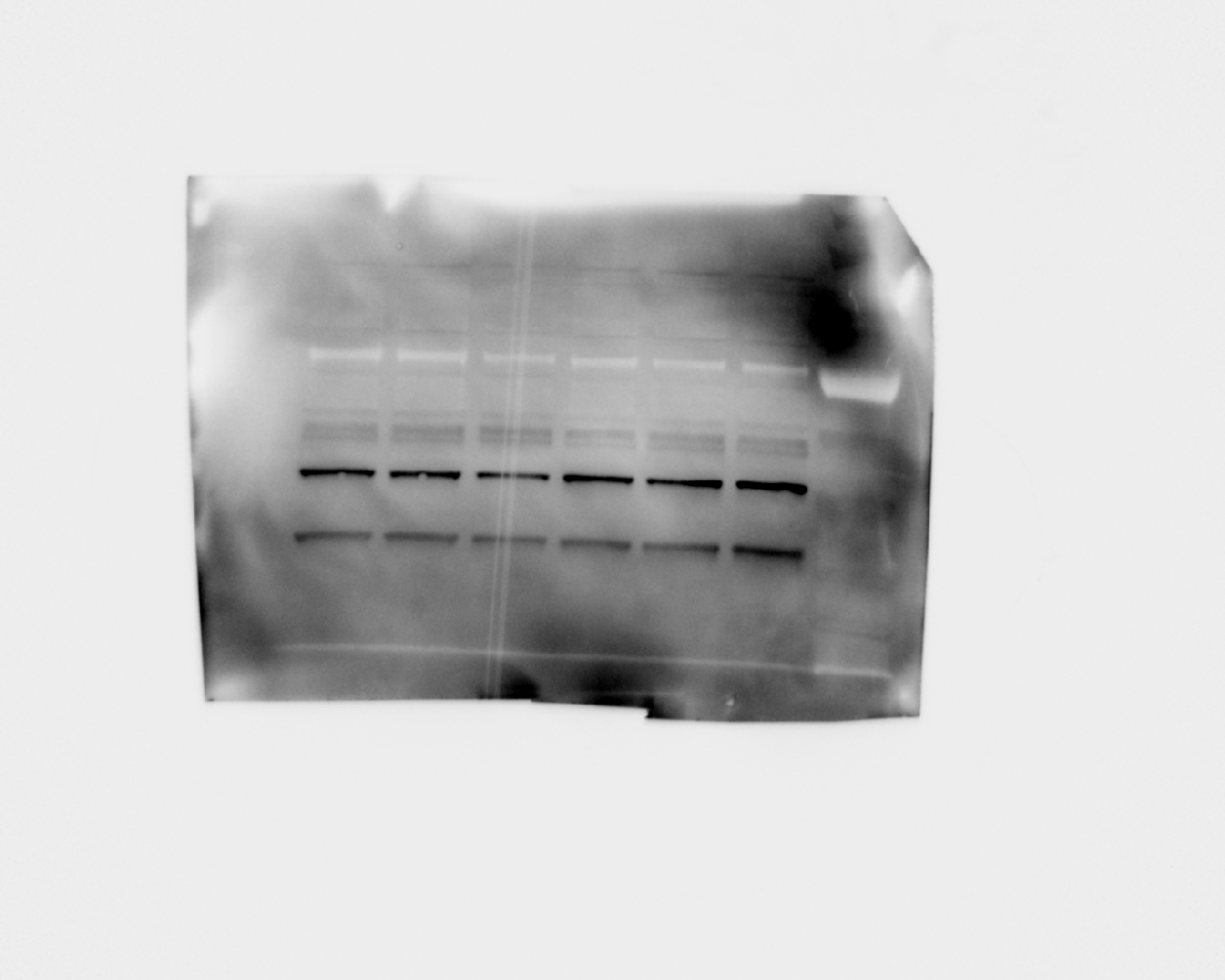

Supplement: Supplementary file 1 [file biomolecules-15-00299-s001.zip › File S2 Uncropped Images/Figure S2-BECN1.tif]

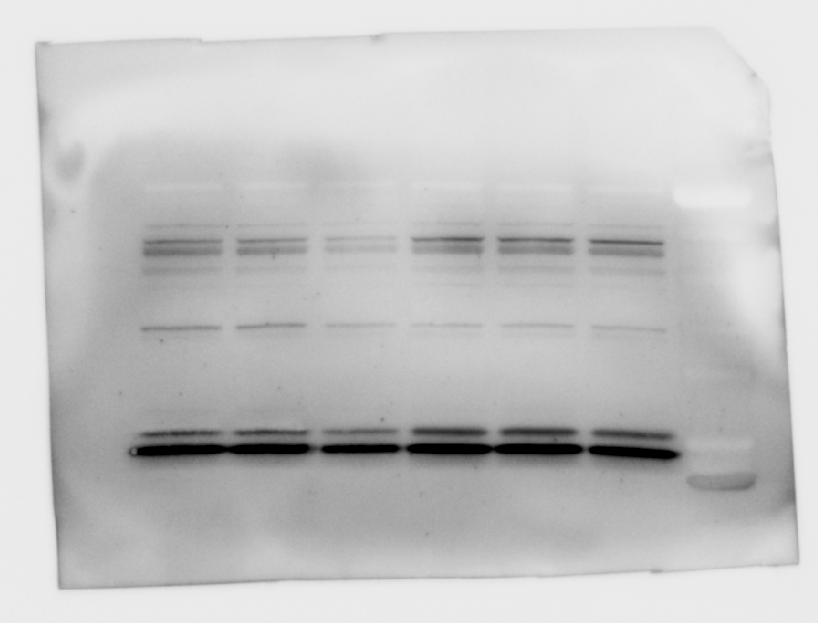

Supplement: Supplementary file 1 [file biomolecules-15-00299-s001.zip › File S2 Uncropped Images/Figure S2-LC3.tif]

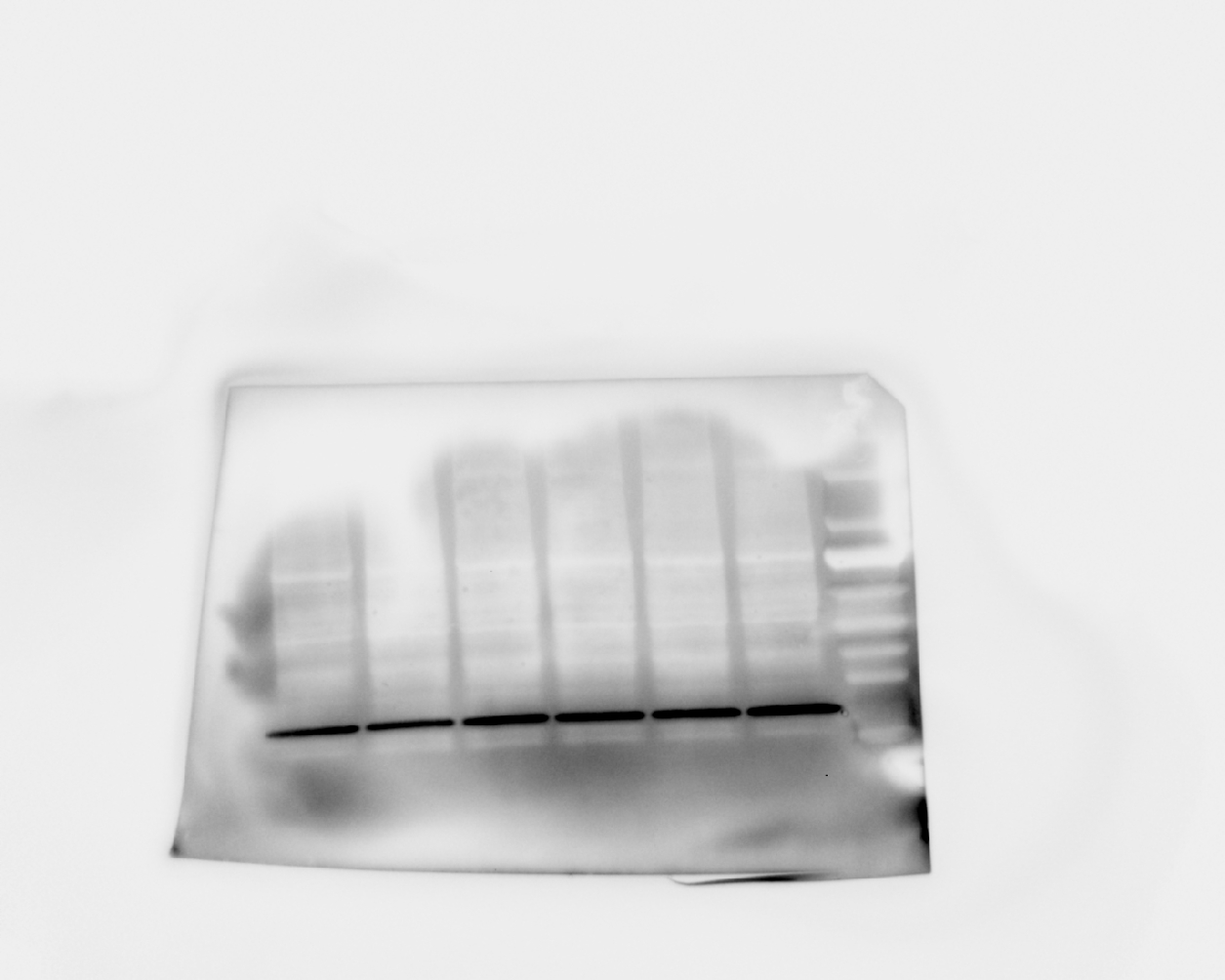

Supplement: Supplementary file 1 [file biomolecules-15-00299-s001.zip › File S2 Uncropped Images/Figure S2-PPIB.tif]

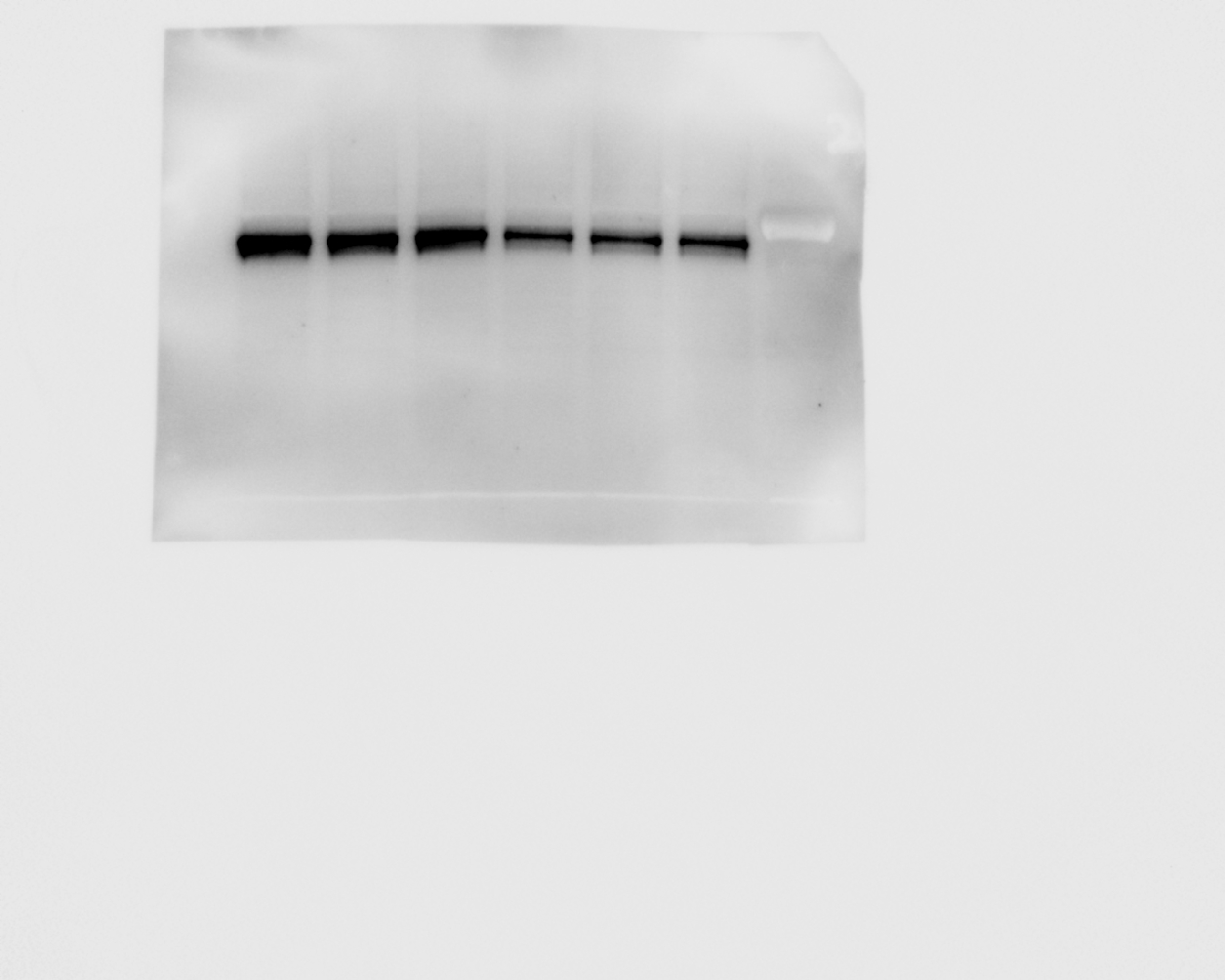

Supplement: Supplementary file 1 [file biomolecules-15-00299-s001.zip › File S2 Uncropped Images/Figure S2-SQSTM1.tif]

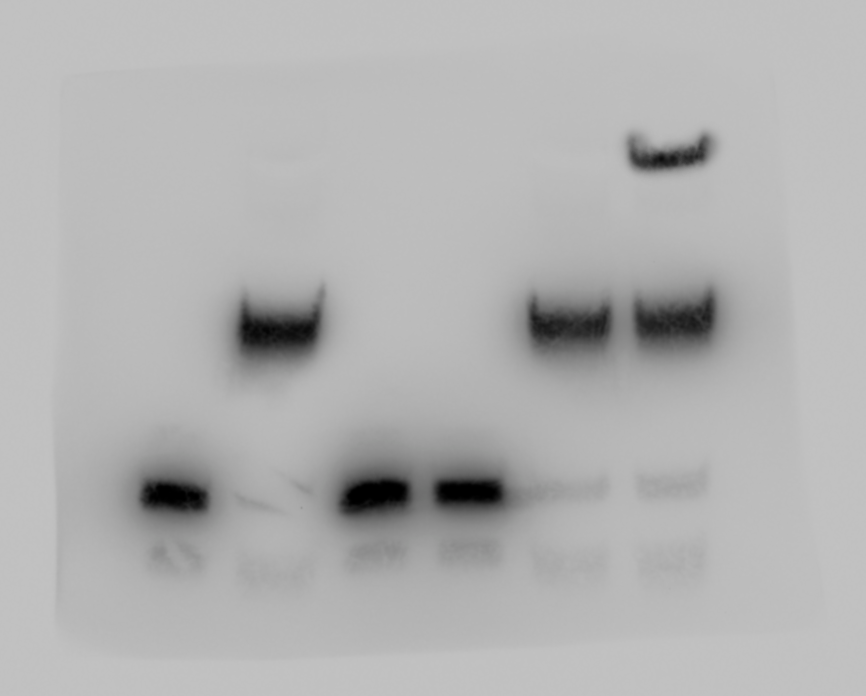

Supplement: Supplementary file 1 [file biomolecules-15-00299-s001.zip › File S2 Uncropped Images/Figure S3.tif]
